# SARS-COV-2 in Argentina

**DOI:** 10.5935/1518-0557.20200071

**Published:** 2020

**Authors:** Stella Lancuba, A. Gustavo Martinez

**Affiliations:** 1 President of Sociedad Argentina de Medicina Reproductiva (SAMeR); 2 Director of CIMER, Argentina; 3 Vice President Red Latinoamericana de Reproducción Asistida (REDLARA); 4 Director of Laboratorio de Fertilis Medicina Reproductiva, Argentina

The SARS-COV-2 pandemic began, as an extraordinary event, on March 3^rd^ 2020. We are facing particular characteristics. Since the outbreak in Wuhan, we have been able to adapt our response from the National Health System toward the virus spread and on the other hand, its localization has become geographically limited to a specific area, only one small part of the totality of our territory.

Since day one, the epidemiologic map has shown more than 80% of cases within the Metropolitan Region of Buenos Aires (The City of Buenos Aires itself, plus 40 adjacent municipalities). This strong penetration within a single geographic area has endured over time and constitutes to this day 90% of cases countrywide. However, in the remaining areas distribution has been markedly heterogeneous, with significantly lower burden. Cordoba, Chaco, Santa Fé and Rio Negro have been most affected.

As members of the Scientific Committee of the Ministry of Health, representatives of both Sociedad Argentina de Medicina Reproductiva (SAMeR) and Red Latinoamericana de Reproducción Asistida (REDLARA) we collaborated with the National Program for Assisted Fertilization of said ministry, elaborating conjoined recommendations in a timely manner so as to coincide with both re-opening of IVF clinics and the implementation of a brand new, COVID-19 aimed informed consent.

On June 8^th^ 2020, REDLARA and SAMeR, together with nine other Latin American societies, signed the position in respect to fertility in times of the COVID-19 pandemic: "**Statement REDLARA and Societies of Latin America**" (REDLARA, 2020).

Simultaneously, SAMeR created the COVID-19 Crisis Committee, to study and fully understand its cytopathic effect on reproductive tissue under current available literature, working together with local healthcare authorities to better understand its epidemiologic aspects. Early measures were taken to actively protect the health of the unit as a whole, physicians and non-physician personnel alike. This was accomplished by taking action to reduce both morbidity and mortality.

According to results from a survey sent by SAMeR to 45 IVF clinics (of which 28 answered), 94% reported having ceased all activities at the beginning of the pandemic. SAMeR recommended to prioritize urgencies, postpone non-urgent activities and to carry out essential activities, based on relayed information from IVF clinic surveys, epidemiologic feedback and the fact that there is a scant rate of complications on the specialty.

Four documents were published during the months of March and April, regarding preventive measures (SAMeR, 2020a). Concurrently, **Covid-19 Risk Management Manual** (SAMeR, 2020b), one of the most relevant features, was published, for use in the reopening of IVF Clinics.

All clinics involved swiftly recognized latent risk, regarding preventive measures to be applied to patients undergoing treatment. All these steps enabled a secure re-opening of clinics with the smallest risk, and continuously working towards optimization of measures to stop viral contagion expansion.

The SARS-COV-2 pandemic bought about profound changes to human activities at a global level. The collaborative spirit and vast professional quality of the members of civil society organizations have integrated in a multi-level information exchange networks. It has aided us in creating new paradigms and new management tools. Always minding our patient’s and our own safety facing the ever-changing viral threat, never setting aside quality and clinic effectiveness.

We have resumed our activities with minimum risk, maximum care and using our newfound knowledge to our advantage. However, we cannot stagger, confirmed SARS-COV-2 cases keep rising, and these first 150 days have taught us greatly. We have been able to manage these uncertain times. We have capitalized on the “golden hour” in veering times. 

Consciousness of individual and collective responsibility is a key aspect as a basic tool to mitigate the effects SARS-COV-2 has had in our lives. The current state of matters enables us to re-think our continent in unity, building strong ties of mutual assistance. This is our message to Latin America.
